# Changes in postoperative opioid prescribing across three diverse healthcare systems, 2010–2020

**DOI:** 10.3389/fdgth.2022.995497

**Published:** 2022-12-06

**Authors:** Jean Coquet, Alban Zammit, Oualid El Hajouji, Keith Humphreys, Steven M. Asch, Thomas F. Osborne, Catherine M. Curtin, Tina Hernandez-Boussard

**Affiliations:** ^1^ Department of Medicine, Stanford University School of Medicine, Stanford, CA, United States; ^2^ Computational & Mathematical Engineering, Stanford University, Stanford, CA, United States; ^3^ United States Department of Veterans Affairs, Palo Alto Healthcare System, Palo Alto, CA, United States; ^4^ Department of Psychiatry, Stanford University School of Medicine, Stanford, CA, United States; ^5^ Department of Radiology, Stanford University School of Medicine, Stanford, CA, United States; ^6^ Department of Surgery, VA Palo Alto Health Care System, Menlo Park, CA, United States; ^7^ Department of Surgery, Stanford University School of Medicine, Stanford, CA, United States; ^8^ Department of Biomedical Data Science, Stanford University, Stanford, CA, United States

**Keywords:** opioid, temporal pattern mining, surgery, postoperative pain, prescribing patterns

## Abstract

**Objective:**

The opioid crisis brought scrutiny to opioid prescribing. Understanding how opioid prescribing patterns and corresponding patient outcomes changed during the epidemic is essential for future targeted policies. Many studies attempt to model trends in opioid prescriptions therefore understanding the temporal shift in opioid prescribing patterns across populations is necessary. This study characterized postoperative opioid prescribing patterns across different populations, 2010–2020.

**Data Source:**

Administrative data from Veteran Health Administration (VHA), six Medicaid state programs and an Academic Medical Center (AMC).

**Data extraction:**

Surgeries were identified using the Clinical Classifications Software.

**Study Design:**

Trends in average daily discharge Morphine Milligram Equivalent (MME), postoperative pain and subsequent opioid prescription were compared using regression and likelihood ratio test statistics.

**Principal Findings:**

The cohorts included 595,106 patients, with populations that varied considerably in demographics. Over the study period, MME decreased significantly at VHA (37.5–30.1; *p* = 0.002) and Medicaid (41.6–31.3; *p* = 0.019), and increased at AMC (36.9–41.7; *p* < 0.001). Persistent opioid users decreased after 2015 in VHA (*p* < 0.001) and Medicaid (*p* = 0.002) and increase at the AMC (*p* = 0.003), although a low rate was maintained. Average postoperative pain scores remained constant over the study period.

**Conclusions:**

VHA and Medicaid programs decreased opioid prescribing over the past decade, with differing response times and rates. In 2020, these systems achieved comparable opioid prescribing patterns and outcomes despite having very different populations. Acknowledging and incorporating these temporal distribution shifts into data learning models is essential for robust and generalizable models.

## Introduction

There are 53 million surgeries performed in the United States every year and the majority of patients experience moderate to severe postoperative pain (1–3). Opioids are often a first-line treatment of postoperative pain and perioperative opioid exposure is considered a gateway to opioid misuse and addiction (4–6). The increased prescription of opioid medications in the United States over the past several decades, including those for postoperative pain, led to widespread dependence and misuse (7). In 2017, more than 191 million opioid prescriptions were written in the United States and these prescription opioids were involved in more than 35% of all opioid overdose deaths (8). Therefore many health care systems and levels of government attempted to promote more judicious prescribing.

In 2010, the Drug Enforcement Administration (DEA) finalized a policy that required 2-factor authentication for electronic opioid prescribing to increase security and improve monitoring and tracking (9). In 2013, the Veterans' Health Administration (VHA) launched the ambitious Opioid Safety Initiative (OSI) to help decrease opioid prescribing practices through data audits and provide feedback (10). In 2016, two federal initiatives targeting opioid prescribing emerged: the Center for Disease Control (CDC) opioid prescribing guidelines (11) and the Surgeon General's “Call to Action” (12). In addition, many states initiated laws, such as the mandatory enrollment in prescription drug monitoring programs and payor rules (13). In addition, prescription drug monitoring programs were developed, such as the Controlled Substance Utilization Review and Evaluation System (CURES), which was mandated in California in 2018 (13). Finally, quality measures were modified to remove opioid incentives (14).

The rush of policies to curtail opioid over-prescribing had to balance the need for adequate pain management. Further complicating the issue is the diversity and non-comparability of populations, both regarding vulnerabilities and pain levels. Often, opioid-prescribing assessments are conducted in single health systems, which limits the generalizability of those insights gained. There is a need to assess the impacts of recent changes in opioid-related policies over a diverse set of patients.

The aim of this study was to characterize opioid prescribing and associated patient outcomes across three diverse healthcare systems over this period of change: 2010–2020. We hypothesized that opioid prescribing at surgical discharge would decrease over the study period and that patient pain outcomes would not change. The study included two closed systems, one with an integrated response to the epidemic [the Veterans Health Administration (VHA)] and another with a comparable population, but less integrated (Medicaid) and an open academic medical center (AMC), serving a different population. The study provides insight on the effectiveness of changing prescribing patterns on patient outcomes.

## Study data and methods

This is a retrospective analysis of postoperative opioid prescribing patterns among adult patients at VHA (01/2010–06/2020), in six diverse Medicaid state programs (01/2016–06/2020), and an AMC (01/2010–06/2020). The years included are based on data availability and data use agreement contracts.

The investigation focused on surgical patients because their pain level is comparable to other opioid indications. Surgeries were categorized using the Clinical Classifications Software (online Supplementary Appendix S1A) and only the most recent surgery was considered. Patients with a length of stay greater than 13 days and patients without continuous healthcare encounters (Medicaid and AMC) were excluded. Finally, patients were excluded with no record of an opioid prescription at discharge. This study received the approval from the institutes' Institutional Review Board (IRB).

To standardize opioid strength assessment, we converted opioid medications to daily Morphine Milligram Equivalents (MME) according to CDC calculation (15, 16). Persistent opioid prescriptions (POP) were defined as a new outpatient opioid prescription between 3 and 6 months after surgery (17). Trends were calculated using the Joinpoint regression algorithm (version 4.9) (18), based on the likelihood ratio test statistic. We identified the best-fitting regression models for each outcome over time and significant changes in the trends. For each slope, the annual percentage change (APC) was calculated.

## Study results

**Figure 1:**
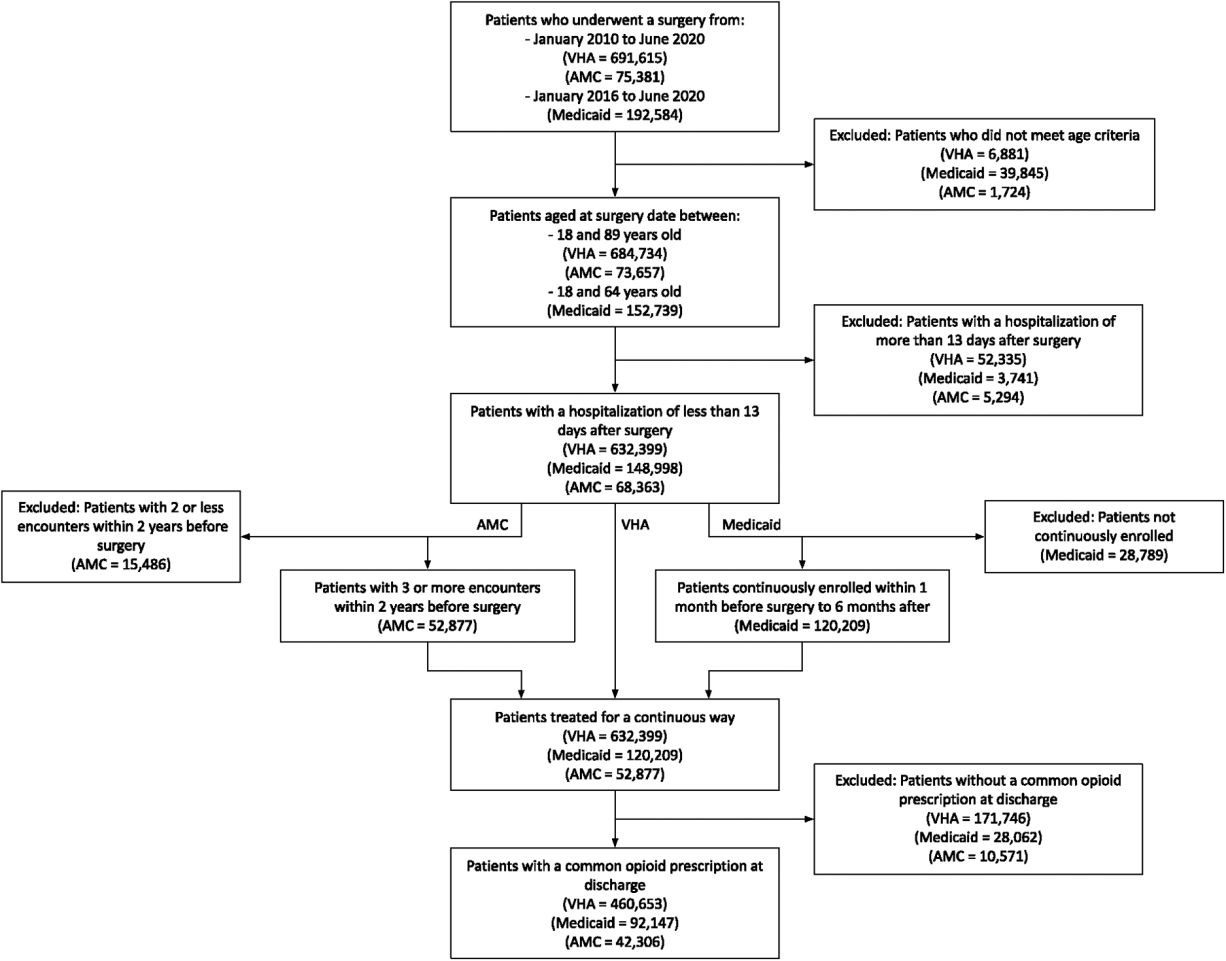
Study flowchart.

A total of 460,653 VHA, 92,147 Medicaid, and 42,306 AMC patients were included in the study (Figure 1). Table 1 shows patient demographic characteristics stratified by population. Overall, VHA prescribed weaker opioid medications at discharge compared to Medicaid and the AMC, with an average MME of 32.2 ± 21.1, 35.5 ± 20.7, and 40.0 ± 19.5 respectively. Yet, both VHA and Medicaid had significantly higher rates of POP compared to the AMC (20.5%, 24.6%, 12.2%, respectively).

**Table 1:** Demographic and clinical characteristics of surgical patients stratified by healthcare system^+^.

		VHA	Medicaid	AMC
N		460,653	92,147	42,306
Age, mean (SD)		60.8 (12.8)	41.4 (12.5)	58.2 (16.1)
Gender, *n* (%)	Female	43,282 (9.4)	64,870 (70.4)	23,543 (55.6)
	Male	417,371 (90.6)	27,228 (29.6)	18,763 (44.4)
Ethnicity and Race, *n* (%)	Hispanic	28,147 (6.4)	N/A	5,997 (14.3)
	Non-Hispanic Asian	2,295 (0.5)		5,275 (12.6)
	Non-Hispanic Black	75,922 (17.4)		1,418 (3.4)
	Non-Hispanic Other*	7,293 (1.7)		3,613 (8.6)
	Non-Hispanic White	323,223 (74.0)		25,651 (61.1)
Insurance payor at surgery date, *n* (%)	Medicaid	0 (0.0)	92,147 (100.0)	3,282 (8.7)
	Medicare	0 (0.0)	0 (0.0)	16,029 (42.7)
	Other	460,653 (100.0)	0 (0.0)	3,256 (8.7)
	Private	0 (0.0)	0 (0.0)	14,981 (39.9)
Pre-surgery pain score^†^, mean (SD)		4.8 (3.5)	N/A	2.5 (2.9)
Surgery type, *n* (%)	Appendectomy	11,278 (2.4)	5,400 (5.9)	954 (2.3)
	CABG	17,390 (3.8)	867 (0.9)	825 (2.0)
	Colorectal resection	18,508 (4.0)	945 (1.0)	2,306 (5.5)
	Distal radius fracture	5,155 (1.1)	2,276 (2.5)	621 (1.5)
	Lysis peritubal adhesions	5,851 (1.3)	1,905 (2.1)	1,277 (3.0)
	Hysterectomy (vaginal/abdominal)	6,756 (1.5)	12,361 (13.4)	3,078 (7.3)
	Inguinal and femoral hernia repair	141,176 (30.6)	11,530 (12.5)	3,050 (7.2)
	Knee replacement	51,413 (11.2)	4,119 (4.5)	4,673 (11.0)
	Oophorectomy	4,408 (1.0)	8,255 (9.0)	1,245 (2.9)
	Other hand	4,016 (0.9)	499 (0.5)	282 (0.7)
	Partial excision bone	43,658 (9.5)	4,903 (5.3)	3,885 (9.2)
	Spinal fusion	19,684 (4.3)	4,295 (4.7)	4,655 (11.0)
	Fracture of hip and femur	5,390 (1.2)	1,191 (1.3)	1,373 (3.2)
	Fracture of lower extremity	17,388 (3.8)	7,500 (8.1)	1,449 (3.4)
	Cholecystectomy	41,743 (9.1)	18,646 (20.2)	3,102 (7.3)
	Laminectomy	24,234 (5.3)	4,365 (4.7)	3,552 (8.4)
	Mastectomy	2,294 (0.5)	1,120 (1.2)	1,772 (4.2)
	Open prostatectomy	23,125 (5.0)	201 (0.2)	2,128 (5.0)
	Thoracotomy	17,186 (3.7)	1,769 (1.9)	2,079 (4.9)

Center (AMC). NOTES: AMC, academic medical center; VHA, veterans health administration; N/A, not available; CABG, coronary artery bypass graft.

^+^ Characteristics obtain at time of surgery.

* Other race category includes Pacific Islander and Native American.

^†^ Pre-surgery pain score is defined as the average pain score within 30 days before surgery.

**Figure 2:**
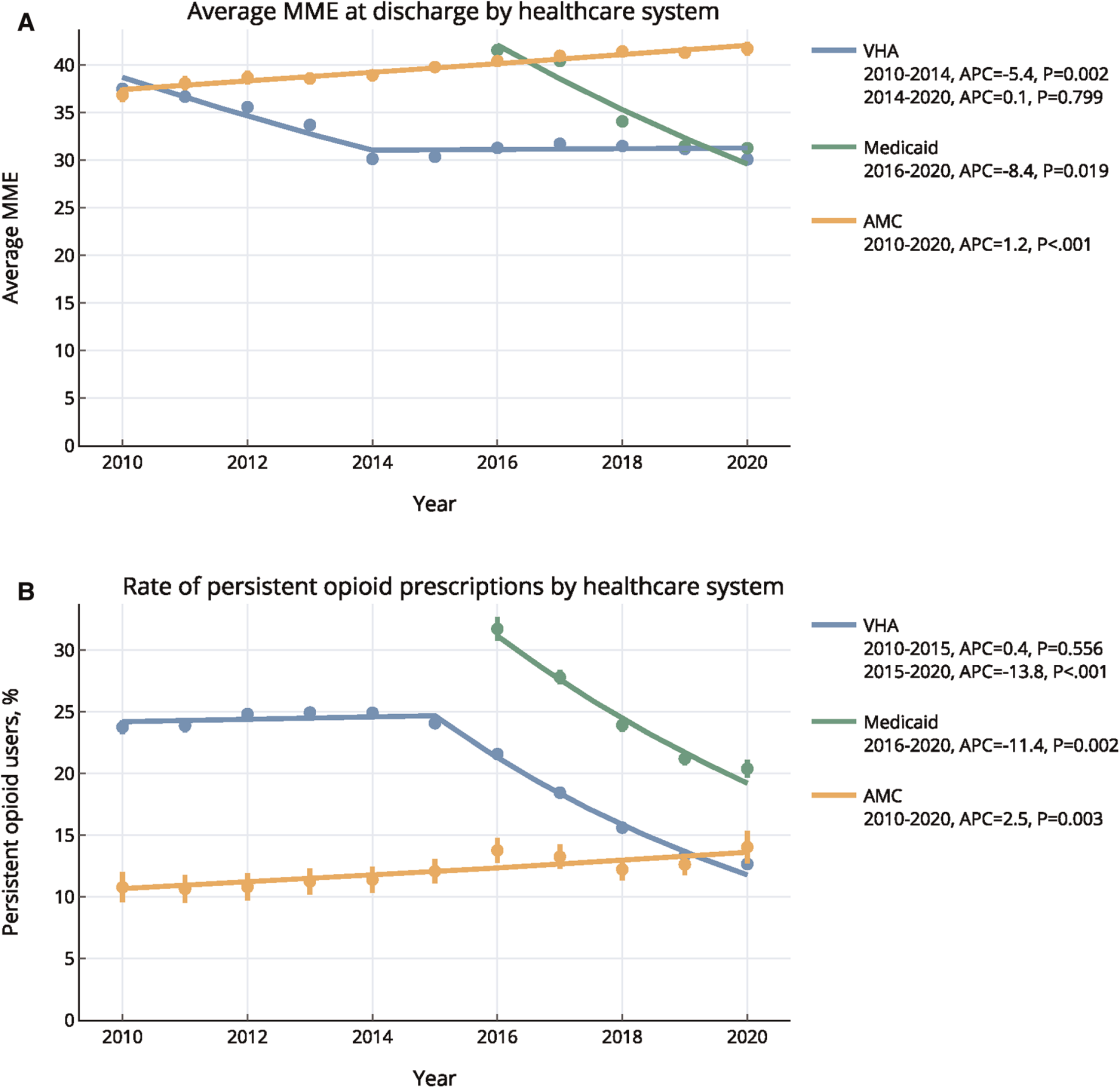
Annual trends of (**A**) opioid prescribing and (**B**) persistent opioid prescriptions in three healthcare systems, 2010–2020 *error bars indicate 95% confidence intervals. Trends were calculated using the ioinpoint regression algorithm. VHA, veterans health administration; AMC, academic medical center; MME, morphine milligram equivalent.

At VHA, the average MME prescribed at discharge decreased from 37.5 in 2010 to 30.1 in 2014 (APC: 5.4%, *p* = 0.002) and remained constant at 31.2 thereafter (*p* = 0.799) (Figure 2A). The average discharge MME in Medicaid decreased from 41.6 in 2016 to 31.3 in 2020 (APC: 8.4%, *p* = 0.019). In the AMC, average MME increased from 36.9 in 2010 to 41.7 in 2020 (APC: 1.2%, *p* < 0.001).

From 2010 to 2015, the rate of POP for VHA remained constant (24.4%; *p* = 0.556), and decreased significantly after 2015 (APC: 13.8%, *p* < 0.001) to reach a rate of 12.7% in 2020 (Figure 2B). For the Medicaid cohort, the rate of POP decreased significantly from 31.7% in 2016 to 20.4% in 2020 (APC: 11.4%, *p* = 0.002). Finally, the rate of POP for AMC increased significantly, rising 2.5% annually from 10.8% in 2010 to 14.0% in 2020 (*p* = 0.003).

Pain scores were available for VHA and AMC. From 2010 to 2020, the average 3-week postoperative pain score decreased from 3.0 to 2.6 for VHA (*p* = 0.004), while remained constant at AMC (avg: 2.1; *p* = 0.068). Other outcomes, such as 30-day readmissions and emergency visits, remained relatively constant over the study period in all healthcare systems (online Supplementary Table S2A).

## Discussion

Multiple policies directed at opioid prescribing patterns have been initiated over the last decade. Understanding the temporal changes of opioid prescriptions and related outcomes in the real-world setting is important for future policy initiatives. We found VHA rapidly decreased average MMEs prescribed at discharge early in the study period and Medicaid followed soon thereafter. In contrast, average MME slightly increased at the AMC. Correspondingly, persistent opioid prescription (POP) rates significantly decreased at VHA and Medicaid, while rates at the AMC slightly increased. However, rates of POP at the AMC started significantly lower than the other two closed systems. Although MME decreased at VHA and Medicaid, patient outcomes remained constant, including pain scores at VHA.

These findings demonstrate important changes in provider prescribing behavior and opioid-related adverse events. Previously, providers were strongly encouraged to minimize pain and opioid prescriptions dramatically increased (19). Here, we demonstrate a pendulum effect, with providers greatly reducing opioid strength, likely in response to policies promoting judicious prescribing, similar to other studies focused on chronic pain. Both VHA and Medicaid had high rates of POP at the start of the study, highlighting the opioid problem in these populations. In 2013, the VHA implemented the OSI program to address to the opioid epidemic. This program was multipronged, targeting education, risk mitigation, pain management and addiction arms (20). The VHA informatics infrastructure allowed for the system to follow individual prescribing provide benchmarks for providers and facilities across the country. Medicaid opioid prescriptions also decreased in 2016, following the multiple state and federal incentives. However, previous studies analyzing opioid prescriptions prior to 2016 in the Medicaid population have shown either consistent or increased opioid prescriptions before 2015 (21–23). In the AMC where individual providers are generally less regulated, MME prescribing increased over the study period. This work suggests providers are responsive to changing landscapes and polices, especially within the tighter controls in closed systems.

Even as opioid prescriptions strengths were reduced, other patient outcomes did not significantly change, as measured by 3-week postoperative pain scores, 30-day readmissions, and emergency visits. This work supports policies reducing opioid consumption, at least from the extremely high levels in 2010 (24). As opioid prescribing declines, proposals to further reduce use must ensure that patient outcomes continue to be maintained, with focus on to avoiding a decline in quality of life due to avoidable pain (25).

Our study has limitations. This is an observational retrospective study and cannot be interpreted as causal, although our findings were robust and represent large population-based cohorts. As we limit prescription data to that which is associated with surgeries, we provide a limited view to opioid prescribing and utilization dynamics. However, as surgery is considered a gateway to prescription opioids (6), this work provides important insights into an important pathway to initial opioid exposure. We could not confirm whether additional prescriptions were related to the index surgery. However, we exclude patients with a second surgery during the follow-up period, in an attempt to mitigate this limitation. In addition, we do not know if the patient used the prescribed medication, therefore the overall POP rates could be erroneous. It is possible that some patients may be represented in multiple service groups (e.g., VHA and AMC) and as data are de-identified we are not able to identify patients in multiple groups, however this number is likely small relative to the overall cohort sizes. Finally, results from three specific populations may not be generalizable to all.

## Conclusion

In this population-based study, we found significant decreases in opioid prescribing patterns in both closed systems (VHA and Medicaid) and corresponding improvements in patient outcomes. The AMC had different trends, likely reflecting the stark difference in patient populations and underlying medical conditions. These temporal shifts will affect the performance and generalizability of associated AI technologies. This work provides insight into the effect of federal initiatives targeting opioid reduction strategies, and highlights reduced postoperative opioid prescribing does not impair patient outcomes. Further research is needed to confirm these findings.

## Data Availability

The data analyzed in this study is subject to the following licenses/restrictions: The data used in this study contain patient identifiers therefore they are not publicly available. Requests to access these datasets should be directed to boussard@stanford.edu.
