# Supplementary material for: Changes in postoperative opioid prescribing across three diverse healthcare systems, 2010–2020
Source: Front Digit Health. 2022 Dec 6;4:995497. doi: 10.3389/fdgth.2022.995497 (PMC9763443; doi:10.3389/fdgth.2022.995497)
Supplement: Supplementary file 1 [file Table1.pdf]

Supplemental Material

eTable 1: Type of surgical procedures included in the study with corresponding CCS procedure category

Surgery types	Multi-Level CCS - Procedures
Appendectomy	9.12
Coronary artery bypass graft (CABG)	7.2
Colorectal resection	9.1
Distal radius fracture*	14.3
Excision; lysis peritoneal adhesions	9.22
Hysterectomy; abdominal and vaginal	12.5
Inguinal and femoral hernia repair	9.17
Knee replacement*	14.7
Oophorectomy; unilateral and bilateral	12.1
Other hand*	14.15
Partial excision bone	14.1
Spinal fusion	14.11
Treatment; fracture or dislocation of hip and femur*	14.3
Treatment; fracture or dislocation of lower extremity*	14.3
Cholecystectomy and common duct exploration	9.16
Laminectomy; excision intervertebral disc	1.3
Mastectomy	3.3
Open prostatectomy	11.2
Thoracotomy	6.8

Source/Notes: SOURCE Procedures codes are from the Clinical Classifications Software. NOTE

Abbreviations: CCS, Clinical Classifications Software.
